# Short term efficacy and safety of PD-1 inhibitor and apatinib plus S-1 and oxaliplatin as neoadjuvant chemotherapy for patients with locally advanced gastric cancer

**DOI:** 10.1097/MD.0000000000040572

**Published:** 2024-11-15

**Authors:** Yunchuan Tang, Li Dai, Zhiqin Wang, Meifeng Zhang, Haitao Xie, Yunshan Yang, Yongjin Zhou, Zhiqiang Yan, Haibin Wang, Hongxin Yang, Lei Zhang, Tong He, Jiaju Chen, Guanghai Wang, Xiangren Jin, Qian Wang

**Affiliations:** a Department of Gastrointestinal Surgery, Affiliated Hospital of Guizhou Medical University, Guiyang, China; b Guizhou Medical University, Guiyang, Guizhou Province, China.; c The Fourth Department of Medical Oncology, Harbin Medical University Cancer Hospital, Harbin, China; d Department of Outpatient Clinic, Affiliated Hospital of Guizhou Medical University, Guiyang, China

**Keywords:** apatinib, carrelizumab, gastric transcatheter chemoembolization, locally advanced gastric cancer, sintilimab

## Abstract

Surgical resection is the cornerstone of treatment for locally advanced gastric cancer (LAGC). Hence, downstaging of the tumor with neoadjuvant therapy is critical for R0 resection and prolongs the overall survival. Data from related studies are lacking, and the literature is scarce. Therefore, a single arm-study was performed on PD-1 inhibitor and apatinib plus S-1 and oxaliplatin as neoadjuvant chemotherapy for patients with LAGC. The findings are expected to serve as a reference for neoadjuvant therapy for LAGC. We assessed 130 LAGC patients using PD-1 inhibitor, apatinib plus S-1, and oxaliplatin as neoadjuvant chemotherapy from January 2021 to October 2022. A total of 104 patients received gastric transcatheter chemoembolization (GTC). The primary endpoint was the rate of clinical complete response, pathological complete response, and safety, while the secondary endpoints were the R0 resection rate and objective response rate of the disease and the disease control rate. A total of 130 patients completed the clinical assessment, of which 6 patients (4.6%) achieved clinical complete response, 87 patients (66.9%) achieved partial response, 30 patients (23.0%) achieved stable disease, and 7 patients (5.5%) experienced progressive disease. The overall response rate was 71.5% (93/130), and the disease control rate was 94.5% (123/130). A remarkable downstaging effect was observed in this study. Downstaging of the T stage and N stage was achieved in 71.5% and 80% of the patients, respectively, which translated into a high R0 resection rate. The findings revealed that 125 patients underwent R0 resection, and the R0 resection rate was 96.1%. According to the observed results, 21.6% of the patients achieved pathological complete response after neoadjuvant chemotherapy. Gastric transcatheter chemoembolization in the first cycle of neoadjuvant therapy was beneficial for tumor regression (*P* < .001). All adverse events were relieved and disappeared after symptomatic treatment, and no grade 4 adverse events were noted. PD-1 inhibitor and apatinib plus S-1 and oxaliplatin are safe and effective as neoadjuvant treatment of LAGC. Gastric transcatheter chemoembolization is useful for tumor regression during neoadjuvant therapy.

## 1. Introduction

Gastric cancer (GC) is 1 of the most common malignant tumors worldwide. Many patients are already in the advanced stage at the time of initial diagnosis, and the prognosis of those with locally advanced gastric cancer (LAGC) is poor.^[1]^ The treatment for GC is comprehensive and is based on surgery. After years of research and development, radical gastrectomy with D2 lymph node dissection has become the standard surgical procedure for patients with resectable GC. Despite the standard plan of D2 lymph node dissection plus chemotherapy, the prognosis of patients with LAGC remains poor even after the complete resection of primary tumors and regional lymph nodes.^[2]^ Prospective randomized controlled studies have reported that systemic chemotherapy extends the survival of patients with LAGC. A study observed that nivolumab plus chemotherapy resulted in significant improvements in overall survival (OS) and progression-free survival (PFS) compared with chemotherapy alone in patients with GC.^[3]^ SOX regimen as neoadjuvant chemotherapy is associated with high efficacy, acceptable adverse effect, and increased rate of D2 lymph nodes dissection and R0 resection. Another study found that apatinib combined with SOX was an effective and safe neoadjuvant therapy for LAGC.^[2]^ Forty patients underwent surgery, with an R0 resection rate of 75.0%, a radiologic response rate of 75%, and a T downstaging rate of 36.4%. Such clinical studies have made a breakthrough in the efficacy of LAGC treatment. However, neoadjuvant data from related studies are lacking, and the literature is scarce. From another perspective, these data support the rationale of using PD-1 inhibitor and apatinib plus S-1 and oxaliplatin for treating LAGC.

Similar to the transcatheter chemoembolization (TACE) in hepatocellular carcinoma (HCC), Cheng et.al from Chongqing Medical University performed this treatment program. A case of gastric transcatheter chemoembolization (GTC) followed by PD-1 inhibitor as neoadjuvant therapy for a patient with LAGC was reported, and the patient achieved not only R0 resection but also pathological complete response (pCR).^[4]^ Our department is also actively engaged in GTC combined with targeted therapy and immunotherapy. After GTC, the blood supply to the tumor tissues is blocked, which can induce tumor necrosis and release various antigens. This is similar to TACE in HCC, which enhances the clinical efficacy of PD-1 antibodies. Meanwhile, the secreted angiogenic factors induce antiangiogenic drugs to attack tumor cells. We believe that combined GTC, targeted therapy, and immunotherapy can improve the prognosis of patients with LAGC.

On the basis of preclinical data and early-phase studies, this single arm-study was conducted to evaluate the efficacy and safety of combined PD-1 inhibitor and apatinib plus S-1 and oxaliplatin as neoadjuvant chemotherapy for patients with LAGC at our center. This study was approved by the ethics committee of the Affiliated Hospital of Guizhou Medical University (2020088K-AMD-03).

## 2. Materials and methods

### 2.1. Clinical data of patients

The clinicopathological data of 130 patients treated with a combined PD-1 inhibitor and apatinib plus S-1 and oxaliplatin from January 2021 to October 2022 were analyzed.

#### 2.1.1. Inclusion criteria

Patients aged 18 to 80 years who were diagnosed with GC; Patients whose tumors expressed PD-L1 CPS (Combined Positive Score) ≥ 1; Patients who have not undertaken surgery, radiotherapy, chemotherapy, immunotherapy, or targeted therapy; Patients without distant metastasis; Patients at the clinical stages of cT3 to cT4 and N0 to N3; Patients with no underlying diseases (such as hypertension, diabetes, heart disease, and thyroid disease); ECOG score < 2; Participants were requested to provide their signed informed consent form before they participated in the study and were recruited based on the inclusion/exclusion criteria.

#### 2.1.2. Exclusion criteria

Pregnant or lactating patients; Patients with serious mental illness; Presence of other types of malignant tumors; Presence of acute infectious diseases, immune system-related diseases, and incontrollable systemic diseases; Patients who are allergic to drug components in the treatment plan. All patients provided their signed informed consent.

### 2.2. Neoadjuvant treatment

**Figure 1.**
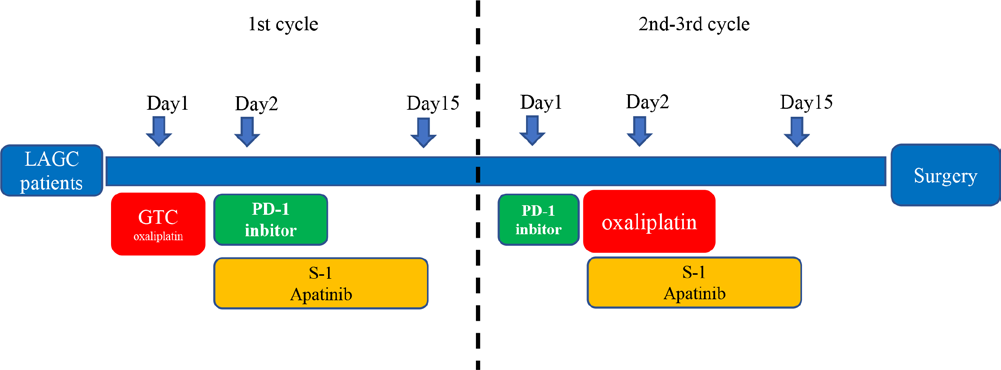
The outline of the treatment process.

Carrilizumab (200 mg) or sintilimab (200 mg) was intravenously administered to the patients on the first day and with oxaliplatin (130 mg/m^2^) on the second day, S-1 was orally administered over days 1 to 14 and the dosage was calculated based on the body surface area (<1.25 m^2^, 40 mg, twice/d. 1.25–1.50 m^2^, 50 mg, twice/day. >1.50 m^2^, 60 mg. twice/d) and apatinib (500 mg/d, days 1–14) over a 21-day cycle (Fig. 1).

### 2.3. Gastric transcatheter chemoembolization

GTC was conducted by experienced gastrointestinal surgeons. Patients did not undergo GTC because of allergic reactions to the contrast agent, high creatinine levels, or unwillingness. According to the study of Cheng et.al,^[4]^ oxaliplatin (100 mg/m^2^) was used as an arterial chemotherapy and lipiodol (10 mL) mixed with oxaliplatin (2 mL) was used for embolism.

### 2.4. Endpoints and assessments

Preoperative efficacy was assessed after 2 to 3 cycles. According to RECIST 1.1,^[5]^ Overall response rate (ORR) is the proportion of patients with clinical complete response (CR) and partial response (PR), while DCR (disease control rate) is the proportion of patients with CR, PR, and stable disease (SD).

Patients were assessed by the Multi-Disciplinary Treatment (MDT) team after 2 to 3 treatment cycles and registered for surgery when the tumor was downstaging or the tumor was not significantly altered despite the 3 cycles of treatment or if the patient had decided to undergo surgery after 2 treatment cycles. Surgery was performed by the chief doctor of gastric surgery. A total of 130 patients underwent laparoscopic radical surgery for GC.

The pathological response was evaluated in patients who received surgery with reference to the Japanese classification of gastric carcinoma and was classified from grade 0 to grade 3.^[6]^ The main outcome measure is pCR and the safety of perioperative treatment, the incidence of adverse events of this combination regimen was observed, which were graded as 0 to 4 with reference to the grading scale of drug toxic reactions (CTCAE 5.0). The secondary outcomes were the R0 resection rate, ORR, and DCR of the disease.

### 2.5. Statistical analysis

SPSS 24.0 statistical software was used for analysis. Descriptive statistics were presented as the mean ± standard deviation. One-way analysis of variance (ANOVA) was employed to determine significant differences among multiple groups, while the Chi-square test was employed to assess differences between the 2 groups. *P* < .05 was considered to indicate a statistically significant difference.

## 3. Results

A total of 130 patients with LAGC were enrolled, and their age was 62 ± 9.3 years. There were 52 (40%) women and 78 (60%) men (Table 1). Of these, 69 (53.1%) patients presented with *Helicobacter pylori* infection. It was observed that 20 (15.3%), 66 (50.7%), and 44 (33.8%) patients had well-, moderately, and poorly differentiated LAGC. All patients (100.0%) were at cT3 to cT4. More detailed information is given in Table 1.

**Table 1:** Baseline demographics data and clinical characteristics.

Items	
Age, yr (range)	62 ± 9.3
Gender	
Male (%)	78 (60%)
Female (%)	52 (40%)
BMI, Mean ± SD (kg/m^2^)	22.5 ± 4.01
ECOG PS, (%)	
0	76 (58.5%)
1	54 (41.5%)
Tumor site	
Cardia	14 (10.7%)
Gastric body	37 (28.4%)
Gastric antrum	79 (60.9%)
Borrman classification	
I type	14 (10.8%)
II to III type	106 (81.6%)
IV type	10 (7.6%)
T stage before neoadjuvant treatment	
cT3	71 (54.6%)
cT4a	59 (45.4%)
T stage after neoadjuvant treatment	
cT1	10 (7.6%)
cT2	61 (46.9%)
cT3	39 (30.1%)
cT4a	20 (15.4%)
N stage before neoadjuvant treatment	
N1	59 (45.3%)
N2	50 (38.4%)
N3	21 (16.3%)
N stage before neoadjuvant treatment	
N0	39 (30%)
N1	65 (50%)
N2	19 (14.6%)
N3	7 (5.4%)
Intraoperative blood loss mean (range), mL	70
Length of hospital stay mean (range), d	10
Helicobacter pylori infection	69 (53.1%)
Tumor differentiation	
High differentiation	20 (15.3%)
Moderately to poorly differentiated or signet ring cell carcinoma	66 (50.7%)
Undifferentiated carcinoma	44 (33.8%)

### 3.1. Effectiveness

**Figure 2.**
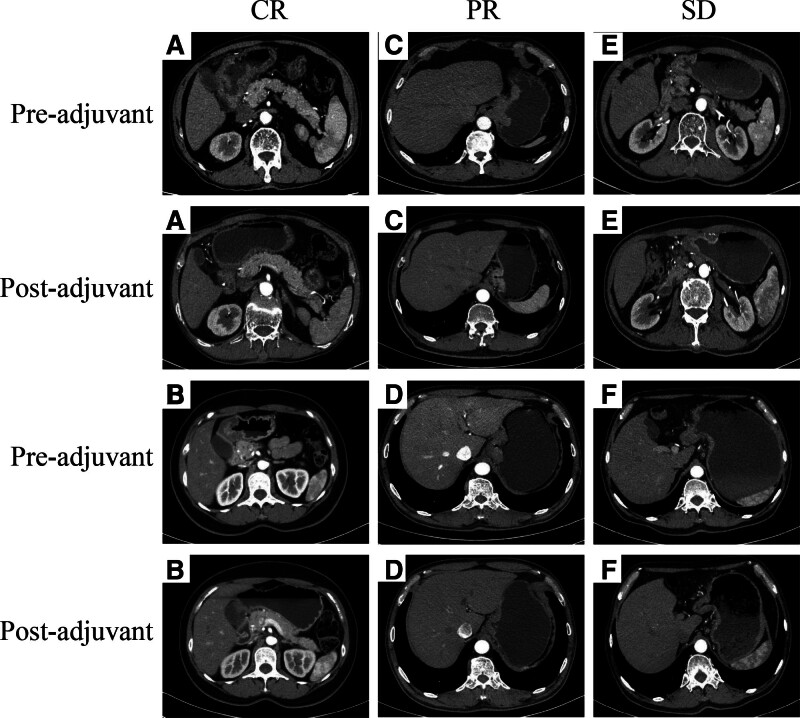
CT scan of the standard patients who achieved CR, PR, and SD after 2 to 3 cycles before and after neoadjuvant treatment. CR = complete response, CT = computed tomography, PR = partial response, SD = stable disease.

After efficacy assessment, 6 patients (4.6%) achieved CR, 87 patients (66.9%) achieved PR, 30 patients (23.0%) achieved SD, and 7 patients (5.5%) achieved PD. The ORR rate was 71.5% (93/130), and the DCR rate was 94.5% (123/130) (Table 2). Compared with the clinical stage before neoadjuvant therapy, 93 (71.5%) patients achieved T downstaging. Moreover, tumor regression was seen in 71.5% of the patients and N downstaging in 104 (80%) patients, which indicated the effectiveness of the neoadjuvant therapy. Ten patients were downstaged from cT3 to cT1. The abdominal computed tomography images showed the standard patients in CR, PR, and SD groups (Fig. 2).

**Table 2:** Clinical response of neoadjuvant chemotherapy.

Response	N (%)
CR	6 (4.6%)
PR	87 (66.9%)
SD	30 (23.0%)
PD	7 (5.5%)
ORR	93 (71.5%)
DCR	123 (94.5%)

Abbreviations: CR = complete response; DCR = Disease control rate; DCR = CR + PR + SD; PD = progressive disease; ORR = objective response rate; ORR = CR + PR; PR = partial response; SD = stable disease.

### 3.2. Adverse events

Adverse events in patients undergoing neoadjuvant therapy were mild and manageable (Table 3). A total of 130 patients completed 2 to 3 cycles of treatment. Common adverse events included leukopenia, anemia, thrombocytopenia, gastrointestinal reactions, elevated aminotransferase level, fatigue, oral mucositis, hypertension, and limb numbness. Approximately 70% of the patients showed hematologic adverse events, and nearly 60% exhibited gastrointestinal reactions. Fortunately, all adverse events were relieved and disappeared after symptomatic treatment, and no grade 4 adverse events occurred.

**Table 3:** Numbers and rates of adverse events in patients undergoing neoadjuvant therapy.

Adverse events	Common terminology criteria for adverse events N (%)			Total
	Grade 1	Grade 2	Grade 3	
Hematologic adverse events				
Leukopenia	43 (33%)	35 (26.9%)	15 (11.5%)	93 (71.5%)
Anemia	43 (33%)	36 (27.6%)	14 (10.7%)	93 (71.5%)
Thrombocytopenia	41 (31.5%)	33 (25.3%)	14 (10.7%)	88 (67.6%)
Non-hematologic adverse events				
Nausea	42 (32.3%)	30 (23.0%)	15 (11.5%)	87 (66.9%)
Vomiting	30 (23.0%)	32 (24.6%)	10 (7.6%)	72 (55.3%)
Elevated transaminase	36 (27.6%)	24 (18.4%)	0 (0.0%)	60 (46.1%)
Abdominal pain	15 (11.5%)	0 (0.0%)	0 (0.0%)	15 (11.5%)
Oral ulcer	22 (16.9%)	15 (11.5%)	0 (0.0%)	37 (28.4%)
Hypertension	13 (10.0%)	0 (0.0%)	0 (0.0%)	13 (10.0%)
Proteinuria	0 (0.0%)	0 (0.0%)	0 (0.0%)	0 (0.0%)
Numbness	6 (4.6%)	0 (0.0%)	0 (0.0%)	6 (4.6%)
Nose bleeding	4 (3.1%)	0 (0.0%)	0 (0.0%)	4 (3.1%)

### 3.3. Surgical outcomes

A total of 130 patients underwent surgery, all of whom underwent D2 lymphadenectomy. R0 resection was not performed in 5 patients (1.5%) owing to progressive disease. Therefore, 125 patients underwent R0 resection, and the R0 resection rate was 96.1%. The median (range) intraoperative blood loss was 70 mL. The median (range) postoperative hospital stay was 10 (5–28) days (Table 1). No readmission, reoperation, or postoperative death within 30 days was observed.

### 3.4. Postoperative pathological response

The pathological response was evaluated in patients who underwent surgery according to the Japanese classification of gastric carcinoma and was categorized from grade 0 to grade 3. After neoadjuvant therapy, 28 patients (21.6%) were rated as grade 3, (pCR), 50 patients (38.4%) as grade 2, 26 patients (20%) as grade 1, and 26 patients (20%) as grade 0 (Table 4).

**Table 4:** Rates of surgical resection, R0 resection, pathological response, and pCR.

Items	Patients (n = 130)
Surgical resection, no. (%)	130 (100%)
R0 resection, no. (%)	125 (96.1%)
Pathological response (TRG grade, no. (%))	
Grade0	26 (20%)
Grade1	26 (20%)
Grade2	50 (38.4%)
Grade3	28 (21.6%)
pCR No (%)	28 (21.6%)

Abbreviations: pCR = pathologic complete response; TRG = tumor regression grading.

Our study included 2 PD-1 inhibitors, namely, carrelizumab and sintilimab. More than half of the patients chose sintilimab because it is much less expensive than carrelizumab. However, the effectiveness of both drugs was similar in this study (Table 5).

**Table 5:** The tumor regression and type of PD-1 inhibitor.

Pathological response (TRG grade, no. (%))	Carrelizumab	Sintilimab	*P*-value
Tumor regression (Y/N)			
Y	33	69	.809
N	11	17	

Abbreviation: TRG = tumor regression grading.

Tumor regression (Y) group contains the patients from TRG 1,2,3 group and the tumor regression (N) group contains patients in the TRG 0 group.

Another special treatment in this study was the GTC, which significantly improved the tumor regression. Compared with 58.5% of the patients who did not undergo GTC, 89.8% of those who underwent GTC experienced tumor regression during the neoadjuvant therapy (Table 6). The findings showed that the effect of GTC was not associated with the tumor location (Table 6).

**Table 6:** Relation of gastric transcatheter chemoembolization (GTC) and tumor regression and location.

	Transcatheter chemoembolization (Y)	Transcatheter chemoembolization (N)	*P*-value
Tumor regression (Y/N)			
Y	80	24	<.0001
N	9	17	
Location			
Cardia	9	5	.1560
Gastric body	22	15	
Gastric antrum	58	21	

Abbreviation: TRG = tumor regression grading.

Tumor regression (Y) group contains the patients from TRG 1,2,3 group and the tumor regression (N) group contains patients in the TRG 0 group.

## 4. Discussion

In China, most patients were LAGC at the time of diagnosis. Thus, early diagnosis should be improved, and an effective method for diagnosing LAGC is urgently needed. People in the Guizhou province have poor awareness of early diagnosis because of their resistance to undergoing gastroscopy, which is a common phenomenon in most areas of China. Once LAGC is diagnosed, all family members have high expectations for surgery. Hence, it is necessary to explore effective treatment methods for LAGC, both for tumor downstaging and improved R0 resection.

In the last 3 years, a series of large prospective studies have been published. For instance, the CheckMate 649 study demonstrated the superiority of the combination of nivolumab and chemotherapy as first-line treatment for LAGC in terms of OS and PFS both in patients with a PD-L1 CPS score of ≥ 5 and ≥ 1 as well as in the general population. In addition, the application of PD-1 inhibitors in the neoadjuvant therapy of tumors, such as melanoma and lung cancer, has yielded encouraging results.^[7]^ Therefore, neoadjuvant therapy of other solid tumors has gained attention, but its application in GC remains in its infancy. Vascular endothelial growth factor receptor 2 (VEGFR2), a major factor responsible for tumor angiogenesis, is an attractive target for novel anticancer therapies.^[8]^ The new small molecule VEGFR inhibitor apatinib, a drug that has been independently developed in China as a third-line treatment option for advanced GC, has achieved reliable efficacy results. The combination of chemotherapy and apatinib has also been used in neoadjuvant chemotherapy for advanced GC with a certain data basis. It provides a theoretical basis for combining chemotherapy with immunotherapy and targeted therapy.

**Figure 3.**
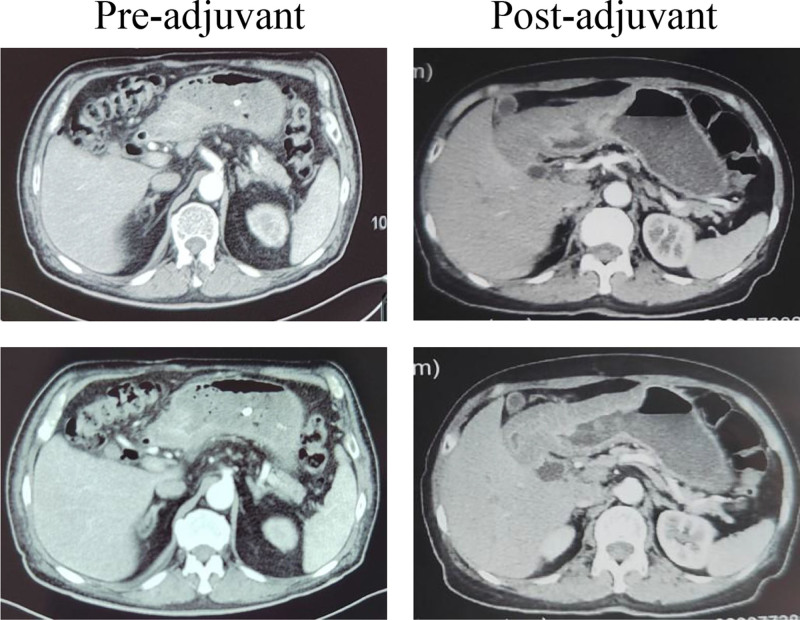
CT scan of the patients who achieved SD after neoadjuvant treatment and the patient classified with a TRG2 pathological response. CT = computed tomography, SD = stable disease, TRG2 = tumor regression grading 2.

A study from Japan showed that the R0 resection rate was 72.1% in the surgery-alone group (106/147) and 80.5% (112/139) in the perioperative chemotherapy plus surgery group^.[9]^ Another study reported an ORR of 68.8% and a DCR of 93.8% in neoadjuvant chemotherapy for patients with LAGC. According to another investigation, neoadjuvant chemotherapy contributed to an 87.1% R0 resection rate and a 2.5% pCR rate in patients with LAGC^.[10]^ A study illustrated that neoadjuvant apatinib plus chemotherapy achieved an ORR of 75%, a T downstaging rate of 36.4%, and an R0 resection rate of 75% in patients with LAGC.^[11]^ Xu et al performed a study on neoadjuvant S-1 plus oxaliplatin combined with a PD-1 inhibitor and apatinib in patients with LAGC.^[12]^ This study is similar to ours, except for the GTC. In their study, 2 (6.7%), 18 (60.0%), and 10 (33.3%) patients achieved CR, PR, and SD, respectively. The ORR and DCR were 66.7% and 100.0%, respectively. In addition, 20% of the patients achieved pCR. In the present study, 93 (71.5%) patients were evaluable for response, 6 (4.6%) patients had CR, 87 (66.9%) patients had PR, and 23 (23.0%) patients had SD. The ORR rate was 71.5%, and the DCR rate was 94.5%. Furthermore, 71.5% of the patients achieved T downstaging and 80% N downstaging, which led to a high R0 resection rate (93.3%). The tumor downstaging rate was satisfactory for both doctors and patients. In terms of postoperative pathological response, 21.6% (28/130) of the patients achieved pCR, which is higher than the reported rate (2% to 20%). In patients with preoperative efficacy evaluation of SD, downstaging of the tumor in the intraoperative period was observed and the postoperative pathological regression grade was 1 to 2, which may be related to the inflammatory edema or tumor fibrosis of the gastric wall after the treatment (Fig. 3).

GTC and TACE are based on the same principle. TACE has been reported to promote T-cell activation via abscopal effects.^[13]^ The tumor necrosis caused by TACE increases the release of tumor-associated antigens.^[14]^ Some studies have also reported encouraging data on the combined use of TACE and tyrosine kinase inhibitor plus PD-1 inhibitor, with an ORR of 75.7% to 84.2% and a DCR of 86.5% to 94.7% in HCC.^[15,16]^ TACE reduces the blood supply to HCC and activates the release of tumor-specific antigens, which enhance the clinical efficacy of PD-1 antibodies.^[17,18]^ Based on the above evidence, GTC could exert a similar effect of enhancing the efficacy of PD-1 inhibitors, but the actual mechanism requires further exploration. In our center, a patient suffered stomach perforation after GTC, which could be related to the cell necrosis of the gastric wall due to a reduction in the blood supply to the gastric wall. This adverse phenomenon reminds us that doctors must pay attention to the patient’s abdominal signs after GTC.

Typically, combined treatment regimens appear to yield better results, but there is also an increase in the number of adverse events. Therefore, safety is the primary consideration in drug combination therapy. A study by Xu et al documented various adverse reactions, including leukopenia (50%), anemia (43.3%), neutropenia (33.3%), fatigue (53%), elevated transaminase level (40.0%), and nausea (23%). Furthermore, grade 3 adverse events, such as leukopenia (6.7%), anemia (3.3%), and neutropenia (3.3%), were noted.^[12]^ In the present study, 15 patients (11.5%) experienced grade 3 adverse events, approximately 11% suffered grade 3 hematologic adverse events, and approximately 50% experienced nausea. The adverse reactions seemed to be higher than those in other studies. Fortunately, no grade 4 adverse events were observed. None of the patients discontinued the treatment or died because of intolerable adverse drug reactions. Also, there were no uncontrollable neoadjuvant-related adverse events. Mostly, adverse events in grades 1 to 2 occurred, which were controllable and did not affect the clinical use of this regimen. Although grade 4 adverse events were not witnessed, they could be attributed to early detection and timely intervention by the doctors. Therefore, doctors should be vigilant to adverse events and respond promptly when patients experience them. We believe that these data provide preliminary evidence for the safety of combined PD-1 inhibitor and apatinib plus S-1 and oxaliplatin as neoadjuvant therapy for LAGC.

In conclusion, combined PD-1 inhibitor and apatinib plus S-1 and oxaliplatin is safe and effective as a neoadjuvant therapy for LGAC with manageable complications, thus providing a novel option for LGAC. GTC is useful for tumor regression during neoadjuvant therapy.

## 5. Limitations

Selection bias could not be ruled out owing to the single arm-design. Nevertheless, the final results showed the effectiveness and safety of PD-1 inhibitor and apatinib plus S-1 and oxaliplatin. In addition, the sample size was not large. A large multicenter randomized clinical trial is needed to confirm the superiority of this regimen in neoadjuvant therapy for LAGC. Another limitation is that the OS rate could not be determined because more than half of the patients did not reach the follow-up time. This data would be provided in our subsequent reports.

## Acknowledgments

The authors of this article would like to acknowledge the patient and relatives for providing adequate case information and for consenting that this work be published.

## Author contributions

**Conceptualization:** Qian Wang.

**Data curation:** Yunchuan Tang, Meifeng Zhang, Haitao Xie, Yunshan Yang, Yongjin Zhou, Lei Zhang, Tong He, Jiaju Chen.

**Formal analysis:** Haibin Wang.

**Funding acquisition:** Zhiqiang Yan, Xiangren Jin.

**Investigation:** Yunchuan Tang, Zhiqin Wang, Hongxin Yang, Guanghai Wang.

**Methodology:** Li Dai.

**Resources:** Tong He, Guanghai Wang, Qian Wang.

**Software:** Yunchuan Tang, Li Dai, Zhiqin Wang, Hongxin Yang, Jiaju Chen.

**Supervision:** Zhiqiang Yan, Haibin Wang, Hongxin Yang, Xiangren Jin, Qian Wang.

**Validation:** Meifeng Zhang.

**Visualization:** Qian Wang.

**Writing – original draft:** Yunchuan Tang.

**Writing – review & editing:** Xiangren Jin.
